# LQT1 patients have augmented response of repolarization dispersion following atropine induced heart rate increase versus healthy controls

**DOI:** 10.14814/phy2.70889

**Published:** 2026-04-26

**Authors:** Pia Dahlberg, Karl‐Jonas Axelsson, Steen M. Jensen, Gunilla Lundahl, Lennart Gransberg, Lennart Bergfeldt

**Affiliations:** ^1^ Department of Molecular and Clinical Medicine, Institute of Medicine, Sahlgrenska Academy University of Gothenburg Gothenburg Sweden; ^2^ Region Västra Götaland, Department of Cardiology Sahlgrenska University Hospital Gothenburg Sweden; ^3^ Heart Centre Umeå University Hospital Umeå Sweden

**Keywords:** atropine, heart rate increase, long QT syndrome, repolarization dispersion, vectorcardiography, ventricular repolarization

## Abstract

The long QT syndrome type 1 (LQT1) is caused by loss‐of‐function mutations affecting the potassium channel current I_Ks_ important for repolarization adaptation at heart rate (HR) increase. We therefore compared changes in three global VR dispersion parameters following a rapid, atropine‐induced HR increase in LQT1 patients versus healthy controls applying Frank vectorcardiography. The adaptation patterns, magnitudes, and changing rates of T amplitude, T area, and the ventricular gradient were assessed during 5 min following injection. Twenty‐one LQT1 patients and 31 controls were enrolled. HR increased similarly within ~23 s. The majority had a tri‐phasic VR dispersion adaptation pattern: a rapid decrease to a minimum, a slower return to a slightly higher level, and a very slow almost horizontal decrease. In the LQT1 group, all three dispersion parameters had a longer lasting rapid phase and larger overshoot reaction, although statistically significant only for T amplitude. In conclusion, LQT1 was associated with an augmented adaptation of global dispersion parameters to a rapid HR increase versus healthy controls. Further studies are needed to determine how these observations relate to arrhythmogenesis and if the atropine test of QT interval and dispersion adaptation can be used in risk assessment on the individual level.

## 1 INTRODUCTION

Congenital long QT syndrome (LQTS) is a hereditary cardiac channelopathy with a prevalence of 1 per 2000. It is associated with an increased propensity for a polymorphic ventricular arrhythmia known as Torsade de Pointes (TdP) ventricular tachycardia. The arrhythmia may terminate spontaneously, cause syncope or via deterioration to ventricular fibrillation, cardiac arrest and death (Amin et al., 2013; Moss & Kass, 2005; Schwartz et al., 2012; Zeppenfeld et al., 2022). The most common subtype, LQT1, is caused by loss‐of‐function mutations in the KCNQ1 gene coding for the membranous slow rectifier potassium channel protein mediating the current I_Ks_. The inadequate outflow of repolarizing positive potassium ions delays ventricular repolarization and typically gives a prolonged QT interval on ECG. Delayed repolarization may cause early after‐depolarization (EAD) and ventricular ectopic beats triggering ventricular tachycardia (Viskin, 1999).

The exact mechanism/s sustaining the TdP in LQTS are debated. However, heterogeneity (aka dispersion) of ventricular repolarization (VR) is thought to be part of the arrhythmia substrate in LQTS, as well as in other ventricular arrhythmias, as proposed more than 40 years ago (Coronel et al., 2009; Coumel et al., 1985; Kuo et al., 1983; Smith & Gallagher, 1980; Viskin, 1999).

In LQT1, arrhythmias typically occur in situations associated with HR increase, and physical exercise is a well‐known scenario of cardiac events, especially swimming (Schwartz et al., 2001). Consequently, we compared several spatio‐temporal measures of VR dispersion before and after exercise testing in LQT1 patients and healthy controls (Dahlberg et al., 2023). The QT, QTpeak, and Tpeak‐end intervals were significantly more prolonged after exercise in LQT1 patients versus healthy controls. There was, however, no difference in the changes of T amplitude, T area, and the ventricular gradient (VG), which are dispersion parameters reflecting global heterogeneities in action potential duration and morphology and change significantly during myocardial ischemia (Bergfeldt, Gransberg, & Lundahl, 2025). The limitation of the exercise study was that motion and breathing induced noise precluded analysis of the recordings obtained during and immediately after exercise. However, Lecocq et al. compared the QT/RR relationship during exercise, catecholamine and atropine administration, and concluded for all three modes, *“These results suggest that physiologic QT‐RR adaptation is mainly under parasympathetic control.”* (Lecocq et al., 1989). Therefore, in parallel with the exercise study, but completed and published a year earlier, we studied QT and QTpeak adaptation during HR increase induced by an intravenous bolus injection of atropine. The quality of the recordings during HR increase was very good, as expected from previous experience (Vahedi et al., 2012). We found that QT adaptation was faster in LQT1 patients versus healthy controls (Dahlberg et al., 2022). But there remained a question regarding the adaptation of T amplitude, T area, and VG during rapid HR increase. Was the adaptation of these parameters different in LQT1 patients versus controls? Faster like QT adaptation? The purpose of this study was to fill this knowledge gap and expand the analysis with a comparison of the adaptation patterns of the three global VR dispersion parameters during rapid HR increase induced by atropine.

## 2 METHODS

The procedure was described in detail earlier (Dahlberg et al., 2022). In the first part, we compared QT and QTpeak adaptation between LQT1 patients and healthy controls. The present study builds on a de novo analysis of the same recordings but focusing on a comparison of the adaptation of T amplitude, T area, and VG.

### 2.1 Study subjects

We recruited LQT1 patients from the cardiogenetic outpatient clinics at two Swedish University Hospitals (Sahlgrenska and Umeå University Hospitals). Eligible for enrollment were otherwise healthy patients with a pathogenic gene variant in KCNQ1 (LQT1), without previous cardiac events such as syncope or cardiac arrest. As reference, an identical analysis was performed of recordings from healthy controls participating in an earlier study where the methodology was the same (Vahedi et al., 2012). In both studies, care was taken to enroll the same proportions of women and men. The study was conducted in accordance with the Declaration of Helsinki and approved by the regional ethics committee in Gothenburg (138–07 and 1021–15). Written informed consent was obtained from all subjects.

### 2.2 Procedure and protocol

The study was performed in a hospital setting. Eight surface electrodes (one serving as reference) for vectorcardiography according to Frank (VCG) were attached (Frank, 1956). A 5‐min baseline VCG was recorded continuously after at least 5 min of supine rest. Then an intravenous bolus injection of atropine (0.04 mg/kg, maximum 5 mg) was administered over 30 s to create a complete inhibition of the parasympathetic influence on the sinus node (Jose & Taylor, 1969). The VCG recording was continued for at least 5 min after atropine injection and the study subjects remained for observation in the clinic for another 2–3 h.

### 2.3 Electrocardiographic recordings and VCG measures

VCG was recorded using a CoroNet II system (Ortivus, Danderyd, Sweden). The signals were sampled at 500 Hz, with an amplifier bandwidth of 0.03–170 Hz. Three orthogonal leads in the X, Y, and Z directions were derived according to the Frank lead system and used to calculate the QRS and T loops. The VCG parameters T amplitude, T area, and VG (aka QRST integral and spatial ventricular gradient, SVG) were used as measures of global VR dispersion (Draisma et al., 2006; Geselowitz, 1983; van Huysduynen et al., 2005). All three parameters have previously been shown to change significantly during an acute anterior ST‐elevation infarction where dispersion of repolarization is caused by differences between ischemic and non‐ischemic myocardium (Bergfeldt, Gransberg, & Lundahl, 2025; Samson & Scher, 1960; Vincent et al., 1977). T amplitude is the maximum T vector inscribed in the T‐vector loop. The T area is the square root of the sum of the squared areas in the X, Y, and Z directions. The VG is the vectorial sum of the QRS area and T area vectors.

### 2.4 Analysis of VR dispersion adaptation

Curves were fitted to individual beat‐to‐beat data recorded during the VR adaptation to increased HR. Applying Microsoft Excel's problem solver, curve fits, based on a combination of exponential and linear equations, of data for patients and controls were obtained as previously described (Axelsson et al., 2021). Before any measurements were performed, all curve fits were scrutinized by the authors to determine if they were technically satisfactory, i.e., curve fits adapted well to data. The adaptation patterns to HR increase of the three dispersion measures were then characterized graphically and analyzed.

**FIGURE 1:**
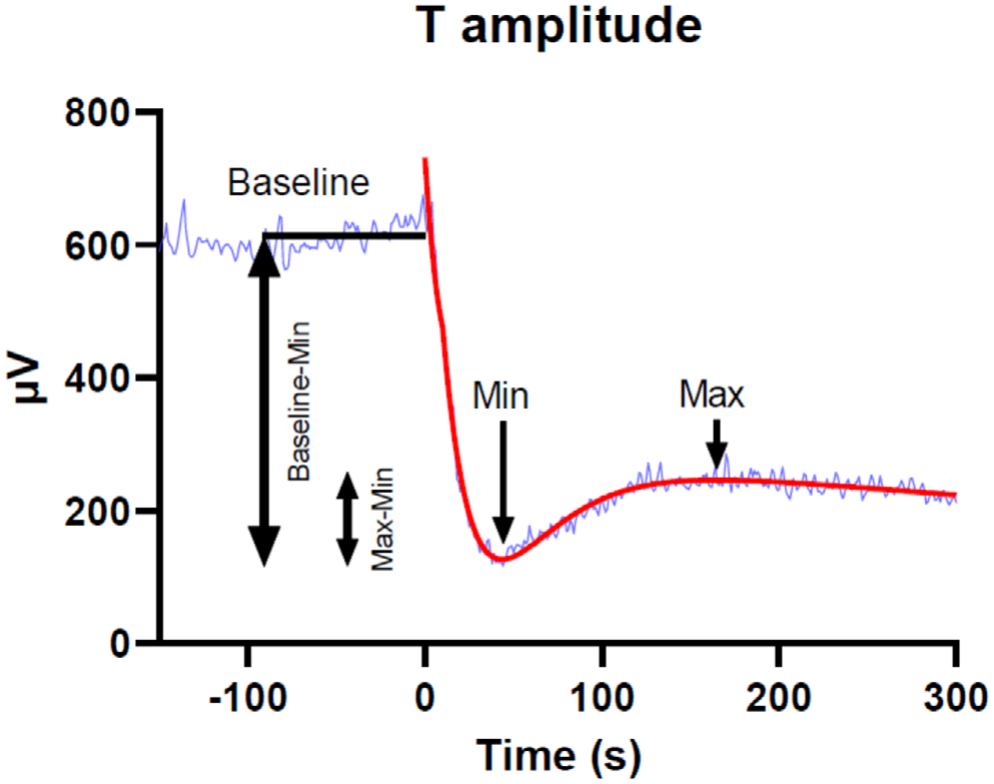
A curve fit (red) to the changes in T amplitude following atropine injection in one LQT1 patient, illustrating the tri‐phasic reaction pattern. The time point 0 denotes the start of HR increase inducing the VR dispersion adaptation to atropine. Min is the minimum value at the nadir of the initial dip. Max is the maximum value after Min. Max‐Min is the overshoot of the reaction during the adaptation to HR increase. The same principles were applied to T area and ventricular gradient.

Most patients and controls had a tri‐phasic adaptation pattern, first a very rapid, nearly linear decrease to a minimum value (Min), secondly a slower nonlinear increase to next extrema (Max), however, at a considerably lower level than the baseline, and thirdly an even slower, nearly linear decrease until the end of the recording. In LQT1 patients and controls with this response pattern, analyses were made on the fitted curves for each of the three VCG parameters to identify the measure points, as illustrated for T amplitude in Figure 1. To reduce the influence of differences in baseline values between study subjects, the change from baseline was considered most adequate. The following measures describe the magnitude of the reaction as well as the time points for the Min‐ and Max‐values: (1) the minimum value (Min) at the nadir of the initial dip, (2) the time from start of HR increase to Min, (3) the Max‐value following the Min‐value, (4) the time from start of HR increase to the Max‐value, (5) the amplitude of the dip in the curve (Max‐ to Min‐value), from here denoted the “overshoot” of the reaction, (6) overshoot ratio, i.e., the proportion of the overshoot relative to the magnitude of the maximum decrease of the parameter from baseline ((Max‐value – Min‐value)/(Baseline‐value – Min‐value)), (7) the rate of change from baseline to Min (Baseline‐Min)/(time from start of HR increase to Min).

**FIGURE 2:**
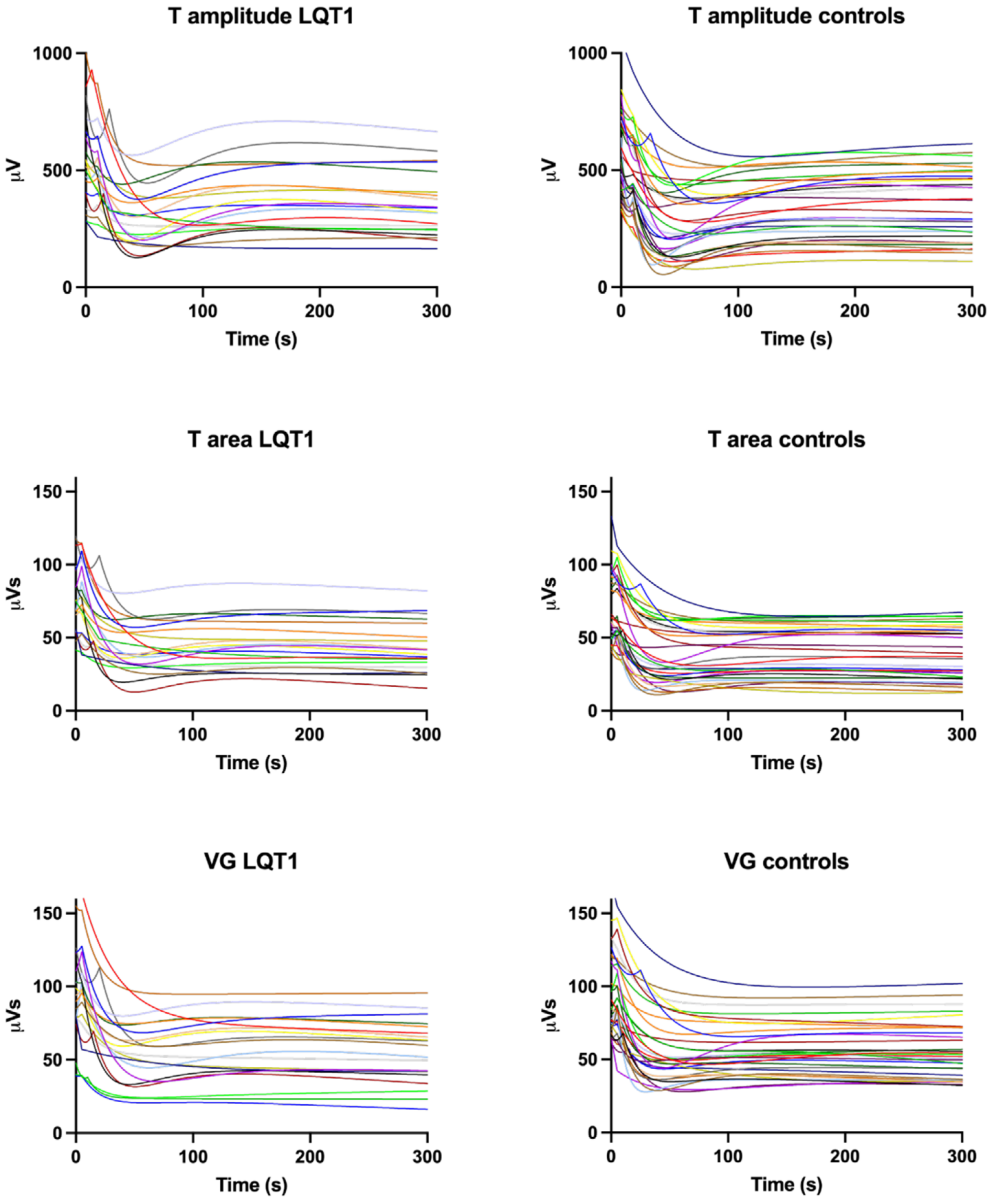
Individual curve fits for the three dispersion parameters, T amplitude, T area, and ventricular gradient (VG), following atropine injection.

Although all patients had a first rapid phase with a decrease of the three parameters, not all patients had the combination of a second nonlinear phase of increase followed by a slow phase of decrease; Figure 2. Therefore, not all patients and controls had definable Min‐ and Max‐values for statistical between‐group comparisons. However, the rate of change for each dispersion measure could be visualized by the derivative of curve‐fits irrespective of the reaction pattern and magnitude. In these graphs the recorded value was averaged for patients and controls and mean (standard deviation) were calculated.

### 2.5 Statistics

Descriptive data are presented as mean (SD). However, non‐parametric tests were used for comparisons to obtain as robust results as possible. The Mann–Whitney *U* test was used for comparisons of patients versus controls. A *p*‐value of <0.05 was considered significant. We used IBM SPSS (version 29), Excel, and GraphPad Prism (version 9.5) for statistical calculations and graphical presentations.

## 3 RESULTS

In total, 21 LQT1 patients and 31 healthy controls were enrolled. Clinical characteristics are presented in Table 1. LQT1 patients were older, had higher mean arterial blood pressure, and longer QTc intervals than controls. Eight patients (38%) were treated with beta‐blockers. As expected, most study subjects experienced dry mouth, accommodation difficulties, and tiredness as a side effect of the atropine injection. There was no arrhythmic adverse event.

**TABLE 1:** Characteristics of study subjects. Data as mean (SD) or numbers (%). Mann–Whitney *U* test was used to compare groups.

	LQT1 (*n* = 21)	Controls (*n* = 31)	*p*‐value
Men/women	11/10	16/15	—
Age (years)	40 (13)	25 (4)	<0.001
Heart rate baseline (bpm)	61 (11)	66 (10)	NS
Heart rate maximum (bpm)	108 (14)	114 (8)	NS
ΔHR maximum–baseline (bpm)	47 (11)	48 (9)	NS
QTcBazett baseline (ms)	456 (29)	387 (21)	<0.001
Body weight (kg)	84 (23)	71 (12)	<0.05
MAP (mm Hg)	95 (11)	73 (9)	<0.001
Beta‐blocker therapy *n* (%)	8 (38%)	0	—

Abbreviations: bpm, beats per minute; MAP, mean arterial pressure.

### 3.1 Heart rate adaptation of repolarization parameters

**FIGURE 3:**
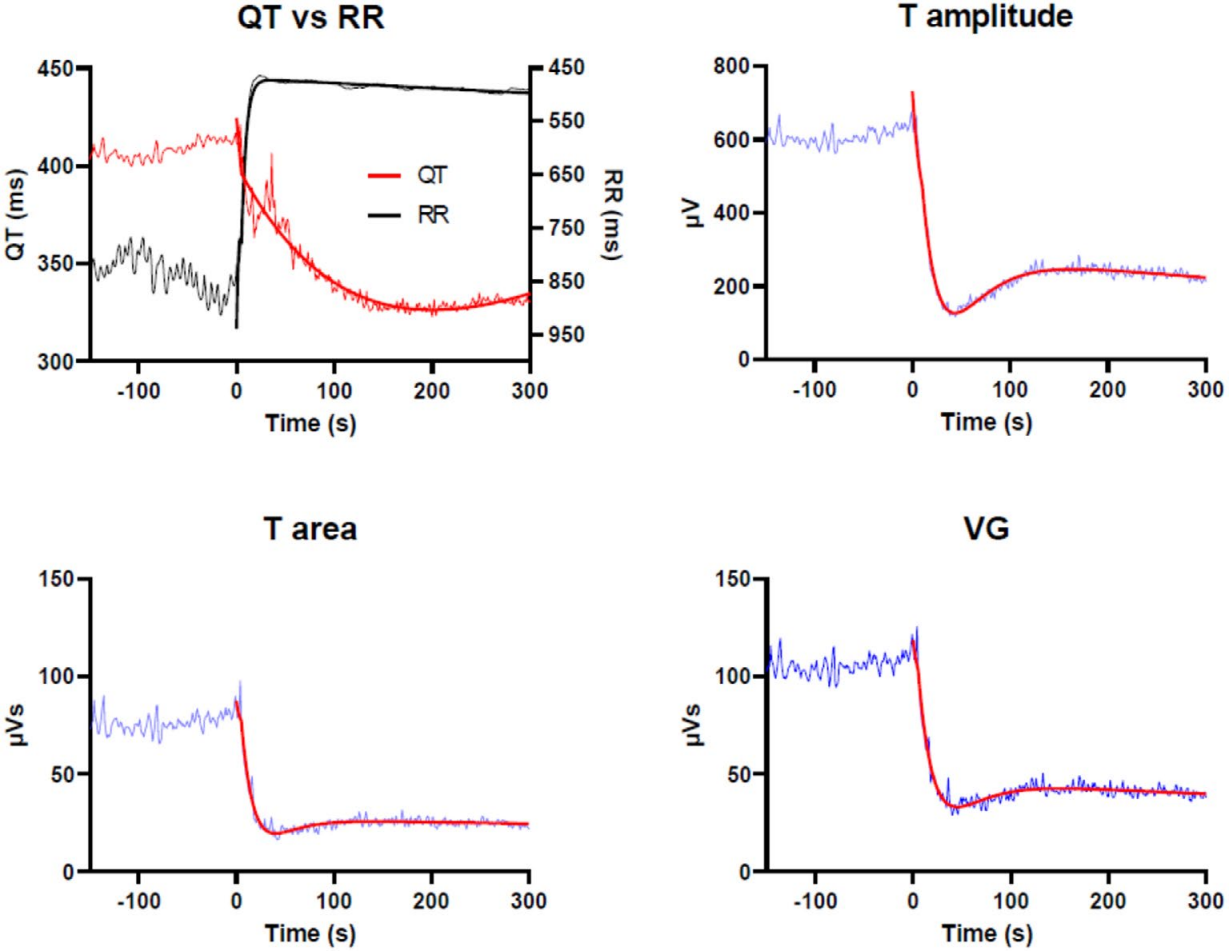
Fitted curves from one individual for reactions following an atropine injection for QT and RR in one panel in the upper left and for the three dispersion measures T amplitude, T area and ventricular gradient (VG) separately. Please observe that the right Y‐axis for the RR‐interval is inversed to enhance visibility, i.e., shorter RR‐interval is higher heart rate. After the injection there is a rapid decrease (dip) in all three dispersion parameters.

The atropine injection induced a prompt rise in HR in all study subjects. Mean HR increased similarly in both groups, from 61 to 108 bpm in LQT1 patients and from 66 to 114 bpm in healthy controls (*p* = 0.7) during on average 22–23 s. All details about the RR intervals and their changes following atropine bolus injection are found in the previous publication on the QT and QTpeak adaptation (see Figure 2, Table 1, Figure S1, Table S1 in this publication) (Dahlberg et al., 2022). Figure 3, from one individual, exemplifies the response patterns for the instantaneous HR (RR interval), QT interval, T amplitude, T area, and VG. There is a very rapid decrease in RR intervals (i.e., HR increase), an exponential decrease in QT with obvious hysteresis, and finally a more rapid adaptation of the three dispersion parameters with an overshoot (which is most pronounced for T amplitude in both groups).

### 3.2 Analyses of adaptation pattern and rate of changes

Figure 2 shows the individual responses for the three VR parameters for all LQT1 patients and controls. Within both groups, the response pattern and magnitude of change varied between individuals in a similar way. Most patients and controls (approximately 57%–67%) had a tri‐phasic adaptation pattern, a first very rapid, nearly linear, decrease to a minimum value (Min), a second slower nonlinear increase to next extrema (Max) at a considerably lower level than the baseline, then a third, even slower, nearly linear, decrease until the end of the recording; Figure 1. In LQT1 patients and controls with this response pattern, measure points could be defined, as illustrated for T amplitude and outlined in Methods, and statistical analyses of between‐group differences were made. Table 2 shows the results from these comparisons for 12–14 patients (57%–67%) versus 18 controls (58%). While the time to Min was slightly shorter for T amplitude than for T area and VG, the reverse was valid for the time to Max. i.e., the dip lasted longer for T amplitude. There were no significant differences between groups. The overshoot in relation to the baseline value (overshoot ratio) was 2–3 times larger for T amplitude than for T area and VG. Furthermore, this proportion was significantly larger in the LQT1‐group, 58 (28) versus 32 (15) %; *p* = 0.003. Regarding T area and VG there were no significant between‐group differences.

**TABLE 2:** Curve fit measures of VR dispersion adaptation in response to atropine. The measures describe the magnitude of the reaction in relation to the baseline values as well as the time points for Min and Max. Data as mean (SD). The measures are described graphically in Figure 1.

	T amplitude (μV)			T area (μVs)			VG (μVs)		
	LQT1 (*n* = 14)	Control (*n* = 18)	*p*	LQT1 (*n* = 13)	Control (*n* = 18)	*p*	LQT1 (*n* = 12)	Control (*n* = 18)	*p*
Baseline	534 (155)	491 (141)	NS	78 (21)	59 (18)	<0.05	93 (21)	81 (18)	NS
Min	298 (128)	217 (126)	NS	41 (19)	27 (14)	<0.05	53 (19)	42 (11)	NS
Max	402 (139)	304 (132)	NS	47 (19)	33 (14)	<0.05	60 (20)	48 (10)	<0.05
Time to Min (s)	48 (16)	49 (14)	NS	56 (25)	52 (18)	NS	54 (12)	57 (22)	NS
Time to Max (s)	171 (21)	181 (33)	NS	155 (31)	156 (43)	NS	155 (32)	168 (58)	NS
Overshoot (Max‐Min)	104 (54)	87 (53)	NS	6.3 (3.5)	5.2 (4.3)	NS	7.2 (3.2)	5.5 (5.3)	NS
Overshoot ratio (Max‐Min)/(Baseline‐Min) (%)	58 (28)	32 (15)	<0.01	19 (9)	16 (9)	NS	19 (10)	14 (9)	NS
(Baseline‐Min)/(time from start of HR increase to Min) (μv/s, μVs/s, μVs/s)	4.8 (2.8)	6.0 (2.5)	NS	0.7 (0.3)	0.7 (0.3)	NS	0.8 (0.4)	0.9 (0.4)	NS

**FIGURE 4:**
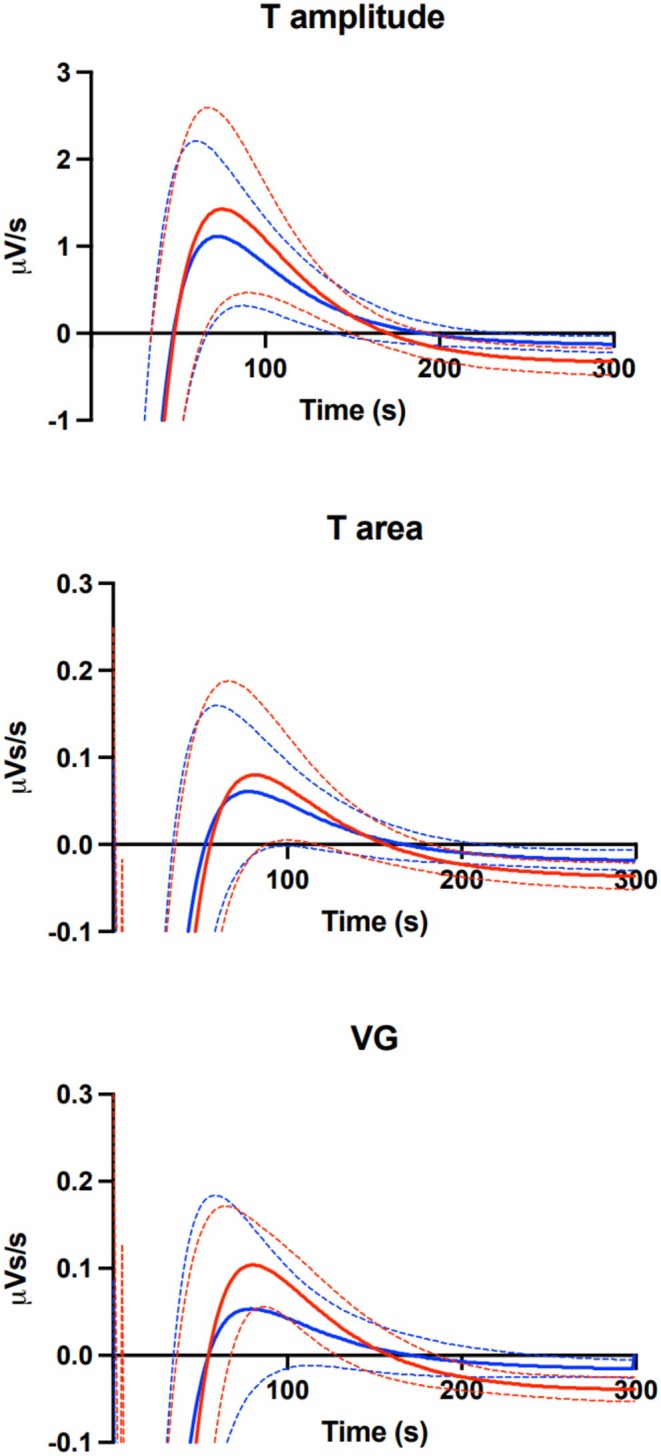
Rate of change in VR dispersion parameters following HR increase induced by an atropine bolus injection. Curves describe the time courses of the derivatives of the adaptation curves for T amplitude, T area and ventricular gradient (VG) for LQT1 patients and controls with the tri‐phasic response pattern as in Table 2. Red lines = LQT1, blue lines = controls, solid lines = mean, dashed lines = standard deviation.

Figure 4 shows derivative curves, i.e., the rate of change at each time‐point for the three dispersion parameters following HR increase for patients and controls with the tri‐phasic response pattern included in Table 2. Red line curves are for LQT1 and blue lines for controls, with means as solid and standard deviations as dashed lines. The area under the derivative curve between zero crossings in Figure 4 represents the magnitude of the overshoot. The average maximum rate of change for T amplitude, T area and VG was 30%–100% higher in LQT1 patients versus controls and the rate of change remained at a higher level.

## 4 DISCUSSION

We compared the adaptation of three global measures of VR dispersion (T amplitude, T area and VG) following a rapid HR increase induced by an atropine bolus injection in LQT1 patients versus healthy controls. Our main findings were: (1) there were no differences in the HR increase, (2) in both groups the VR response pattern was tri‐phasic in the majority, i.e., a rapid almost linear decrease to a dip (overshoot) followed by a nonlinear rebound to a slightly higher level well below baseline, and then a slow almost linear phase over 3–4 min, and (3) the derivative graphs showed that the rate of change and its maximum value were higher and remained at a higher level in LQT1 patients. Further studies are needed to determine how these observations relate to arrhythmogenesis and if the atropine test of QT interval and dispersion adaptation can be used in risk assessment on the individual level.

In an earlier report on the same cohort we showed that although the HR reaction after atropine was similar, LQT1 patients had significantly shorter QT adaptation time (hysteresis) than healthy controls despite a larger change in QT. Both the initial phase reflected by the time constant tau (τ) and the subsequent slower phase reflected by T90 End were shorter in patients (Dahlberg et al., 2022). In the present study based on a de novo analysis of the same material, the patterns of the individual dispersion responses were similar in the two groups and mostly tri‐phasic. There was no significant between‐group difference in the duration or the speed of the initial phase to Min, but the patient group had an augmented overshoot, although statistically significant only for T amplitude, Table 2 and Figure 4. In control systems, a faster response, such as shown for the QT interval adaptation, is usually associated with a higher tendency for overshoot and instability. Furthermore, in a previous study, the short‐term variability (STV) at beat‐to‐beat analyses of several VCG‐based parameters were performed at rest (Vahedi et al., 2012). STV‐QT was on average 115% higher in LQT1 patients versus controls (4.3 vs. 2.0) but even higher in LQT2 patients versus controls (6.3; 215% larger), and STV‐T amplitude was 100% higher in both LQTS types versus controls. The higher STV‐QT and STV‐T amplitude in LQTS patients was observed against a background of less HR variability in LQTS patients, STV‐RR was 37 (LQT1) and 55 (LQT2) % of that of controls. So, at rest there is a higher T amplitude variability or T wave alternans on the mV level in LQT1 (and LQT2) patients despite less RR variability. The augmented response in T amplitude adaptation to HR increase in the present study might also reflect the propensity for VR instability in LQT1 in line with T wave alternans that can be observed with surface ECG (Schwartz & Ackerman, 2013). Taken together, larger VR variability at rest, accelerated QT interval adaptation/abbreviated hysteresis and an augmented response of VR dispersion parameters following atropine induced rapid HR increase seem logical and likely salient functional features in LQT1.

In this context, we would like to emphasize differences between changes in VR during myocardial ischemia on one hand and in LQT1 patients in relation to exercise driven and atropine induced HR increase on the other hand. There is experimental evidence that ischemia increases hysteresis, while it is decreased in LQT1 (Dahlberg et al., 2022; Lauer et al., 2006; Starobin et al., 2007). T area (increase), T amplitude (decrease), and VG (increase) change significantly during acute myocardial ischemia (together with the ST‐segment and T wave morphology parameters), but these three parameters show no changes in LQT1 patients in relation to exercise although HR corrected QT and Tpeak‐end intervals prolonged (Bergfeldt, Axelsson, et al., 2025; Dahlberg et al., 2023). During the acute phase of a myocardial infarction, the HR corrected QT decreased despite a minor but significant decrease in HR (Lingman et al., 2014). Both conditions are associated with increased propensity for polymorphic ventricular arrhythmia, but VR duration and dispersion measures are affected differently, emphasizing VR complexity.

### 4.1 The atropine model – Effects of heart rate increase

According to present knowledge, and as discussed in our study on QT adaptation to atropine induced HR increase, there is a relative lack of parasympathetic innervation of the ventricular myocardium (Dahlberg et al., 2022; Lecocq et al., 1989). The HR increase per se is therefore presumably the most important input to the dynamical dispersion adaptation observed in this study. Atropine, however, not only inhibits the dominant parasympathetic influence on the sinus node at rest but also discloses the minor sympathetic influence (Vahedi et al., 2012). The atropine bolus is followed by an increase in both HR and mean arterial pressure. Secondary effects thereof on the ventricular myocardium can therefore not be ruled out but should be similar for patients and controls, although possibly modified in the 8 LQT1 patients on betablockers. It has been suggested that LQTS patients are more sensitive to sympathetic activity than healthy controls, but the HR response was similar in this study suggesting similar sensitivity at least in the sinus node. According to a post hoc analysis of LQT1 patients with versus without betablocker therapy, there was no difference in the change of RR intervals (Dahlberg et al., 2022).

In the previous exercise study, a goal was to analyze VR adaptation during HR increase and decrease but the motion and breathing induced noise precluded such analysis. The quality requirements were not fulfilled; only the RR intervals could be satisfactorily defined (Dahlberg et al., 2023). The exercise test was in that study performed on an ergometer cycle in the supine position. A thread‐mill test could have been an alternative but not necessarily better from the recording perspective. Another alternative would be rapid standing. The change in body position induces a fast HR increase but also a very complex change in the autonomic nervous system activity due to the redistribution of blood. In addition, the change in body position also takes time and might at least temporarily impair the recording quality. Catecholamine infusion is not a good alternative to atropine in humans. The dose–response to isoprenaline infusion is individually highly variable, necessitating step‐up in infusion rate and thereby slowing the rate of the HR increase. Isoprenaline infusion also results in a similar maximum HR as that following an atropine bolus (Vahedi et al., 2012). Finally, there is a safety issue. Isoprenaline might induce arrhythmia/early after‐depolarizations (EAD) in LQTS patients (Shimizu et al., 1991). We did not observe any arrhythmic adverse events in our LQT1 patients who up to then had no related symptoms. The atropine test allows evaluation of VR measures when HR increases but due to the long half‐life of the atropine effect, evaluation of dispersion at HR decrease was not possible. Recognizing the limitations of the atropine bolus test, we still consider this to be a valuable complement to the exercise test. Together, they provide insights into the VR response to changes in HR. Atrial pacing allows evaluation of VR adaptation to both HR increase and decrease but is rarely an option in LQT1 patients (Axelsson et al., 2021; Bergfeldt, Axelsson, et al., 2025).

### 4.2 Adaptation of VR dispersion, ion channel distribution and arrhythmogenesis

Arrhythmogenesis in LQTS has been investigated in several animal models but the exact mechanism/s behind the polymorphic TdP ventricular tachycardia in LQTS patients remain to be elucidated. It is generally assumed that a combination of a trigger and a substrate is needed for an arrhythmia to occur (El‐Sherif, 2001). The trigger in LQTS is likely ventricular extrasystoles caused by EADs and the substrate functional differences in local refractoriness which create temporary barriers around which an unstable reentry can occur (Brunner et al., 2008; El‐Sherif, 2001; El‐Sherif et al., 1997; El‐Sherif et al., 2017; Liu et al., 2012; Xie et al., 2010).

The tri‐phasic response in VR dispersion was previously found to be a normal physiological phenomenon in response to HR increase also by pacing (Axelsson et al., 2020; Axelsson et al., 2021). We have suggested that this is the result of differences in adaptation time in myocytes in different areas of the ventricles which creates variable temporary ‘gradients’ in VR dispersion during the adaptation to a new HR (Axelsson et al., 2021). This is consistent with animal experiments as well as human findings (Bueno‐Orovio et al., 2012; Bueno‐Orovio et al., 2014; Rosenbaum et al., 1991).

LQT1 is caused by loss‐of‐function mutations in the KCNQ1 gene encoding parts of the potassium channel responsible for the delayed rectifier K+ current I_Ks_ (Amin et al., 2013; Goldenberg et al., 2008; Schwartz et al., 2012). I_Ks_ is of little importance to repolarization in the resting state but is essential to the adaptation of the VR duration to increased HR (Bartos et al., 2015; Jost et al., 2005). In normal physiology, I_Ks_ channels are not uniformly distributed in the ventricles and the density of I_Ks_ in the apex is approximately doubled compared to the base (Szentadrassy et al., 2005). If the density and/or functional characteristics in I_Ks_ already are low in the basal region of the left ventricle (compared to the apex), the impairment of the current in LQT1 would possibly increase the differences in local adaptation time along the existing apico‐basal gradient. But the opposite is also an alternative. A decrease in the apico‐basal or other gradients may result if a HR increase affects the ion channels with rapid action potential adaptation more than those with a slower response. At the nadir of the overshoot (or dip), the dispersion was at its minimum during the 5‐min study period and thus considerably less than at baseline. Some polymorphic ventricular arrhythmias are preventable by overdrive suppression such as after atrioventricular junction ablation (aka His‐ablation) and ventricular pacing (Nowinski et al., 2002). To what extent this therapeutic effect is related to decreased dispersion remains to be elucidated.

So, why do arrhythmias in LQT1 preferentially occur in situations with physical activity such as swimming? Does VR dispersion play any role, and, if so, how? The common concept is that swimming, like most physical activities, is associated with increased HR. The diving reflex might, however, counteract HR increase, as proposed by the results of a Danish study where diving was simulated by face immersion in cold water (Marstrand et al., 2021). Patients with LQT1 and LQT2 reacted with an equally significant decrease in HR and of similar magnitude as controls. Consequently, the HR corrected QT interval increased as expected, but similarly for the three groups. The beat‐to‐beat adaptation of the QT interval was, however, not studied, and neither were any other dispersion measures. Is VR instability in LQT1 patients augmented in this situation because of an autonomic conflict between the tachycardia inducing physical activity and cold shock response on one hand and the bradycardia inducing diving response on the other (Shattock & Tipton, 2012)? An increase in the QT interval would increase the risk for an EAD. And tentatively, the larger the decrease in HR in LQT1 patients at face contact with water during diving, the larger the increase in VR dispersion. This also remains to be elucidated.

### 4.3 Methodological aspects and limitations

The LQT1 patients and the control group were not matched for age, but the proportion of women and men was similar. The differences in age and blood pressure were significant. As discussed above, there is experimental evidence that ischemia increases hysteresis (Lauer et al., 2006; Starobin et al., 2007). Therefore, it seems unlikely that higher age and blood pressure in the LQT1 group would lead to shortened QT adaptation time compared with younger healthy controls; the opposite would be more likely. If the same holds for the adaptation of VR dispersion reflected by T amplitude, T area, and VG is not known.

Due to the individually different response patterns, not all recordings could be used for statistical analyses. Consequently, the numbers are limited, and the results should be interpreted with caution until reproduced. For the derivative data, the average graphs were similar when comparing those based on patients and controls with the tri‐phasic response pattern as in Figure 4 to graphs based on all enrolled patients and controls (not shown) where the graphs, however, were slightly flatter as expected from Figure 2 showing all individual response curves.

## 5 CONCLUSION

A tri‐phasic response in VR dispersion to HR increase is a normal physiological reaction but was augmented in LQT1, especially for T amplitude. The rate of change and its maximum value were higher and remained at a higher level in LQT1 patients. Further studies are needed to provide a deeper mechanistic understanding of how these observations relate to arrhythmogenesis and if the atropine test of QT interval and VR dispersion adaptation can be used in risk assessment on the individual level.

## AUTHOR CONTRIBUTIONS

**Pia Dahlberg:** Conceptualization; data curation; formal analysis; investigation; methodology; visualization. **Karl‐Jonas Axelsson:** Conceptualization; data curation; formal analysis; investigation; methodology; validation. **Steen M. Jensen:** Investigation; methodology. **Gunilla Lundahl:** Conceptualization; data curation; formal analysis; methodology; software; validation. **Lennart Gransberg:** Conceptualization; data curation; formal analysis; methodology; software; validation. **Lennart Bergfeldt:** Conceptualization; formal analysis; funding acquisition; investigation; methodology; project administration; resources; supervision; validation.

## FUNDING INFORMATION

This work was supported by the Swedish Heart and Lung Foundation to LB (20230703) and by grants from the Swedish state under the agreement between the Swedish government and the county councils, the ALF‐agreement to LB (ALFGBG‐1006070). The sponsors had no role in the study design, data collection and analysis, decision to publish, or preparation of the manuscript.

## CONFLICT OF INTEREST STATEMENT

No conflict of interest, financial or otherwise, are declared by the authors.

## ETHICS STATEMENT

The study was conducted in accordance with the Declaration of Helsinki and approved by the regional ethics committee in Gothenburg #1021‐15.

## CONSENT

Written informed consent was obtained from all subjects.

## Data Availability

The participants of this study did not agree to share data with a third party because that is not a required part of the consent according to the Swedish ethics board system. However, some data supporting the findings of this study might be available from the corresponding author upon reasonable request.
